# Biomedical Application of MSCs in Corneal Regeneration and Repair

**DOI:** 10.3390/ijms26020695

**Published:** 2025-01-15

**Authors:** Maria P. De Miguel, Marta Cadenas-Martin, Martha Stokking, Ana I. Martin-Gonzalez

**Affiliations:** Cell Engineering Laboratory, La Paz University Hospital Health Research Institute, IdiPAZ, 28046 Madrid, Spain; cadenasm95@gmail.com (M.C.-M.); marthastokking@gmail.com (M.S.); anaisabelmartin24@gmail.com (A.I.M.-G.)

**Keywords:** cornea, vision loss, mesenchymal stem cells, limbal stem cell deficiency, keratoconus, endothelial defect, stem cell therapy

## Abstract

The World Health Organization estimates that approximately 285 million people suffer from visual impairments, around 5% of which are caused by corneal pathologies. Currently, the most common clinical treatment consists of a corneal transplant (keratoplasty) from a human donor. However, worldwide demand for donor corneas amply exceeds the available supply. Lamellar keratoplasty (transplantation replacement of only one of the three layers of the cornea) is partially solving the problem of cornea undersupply. Obviously, cell therapy applied to every one of these layers will expand current therapeutic options, reducing the cost of ophthalmological interventions and increasing the effectiveness of surgery. Mesenchymal stem cells (MSCs) are adult stem cells with the capacity for self-renewal and differentiation into different cell lineages. They can be obtained from many human tissues, such as bone marrow, umbilical cord, adipose tissue, dental pulp, skin, and cornea. Their ease of collection and advantages over embryonic stem cells or induced pluripotent stem cells make them a very practical source for experimental and potential clinical applications. In this review, we focus on recent advances using MSCs from different sources to replace the damaged cells of the three corneal layers, at both the preclinical and clinical levels for specific corneal diseases.

## 1. Introduction: The Cornea

The World Health Organization estimates that approximately 285 million people suffer from visual impairments, around 5% of which are caused by corneal pathologies [1]. Corneal diseases are a major cause of vision loss, second only to cataracts in overall importance [2]. Approximately 14.5 million people around the world are unilaterally or bilaterally blind due to corneal disease or injury [3,4], with about 2 million new cases diagnosed each year [2,5]. From a socioeconomic point of view, vision loss is among the most feared diseases/disabilities [6]. Depression is estimated to be twice as prevalent among people suffering sight loss, and 75% of blind Europeans suffered chronic unemployment [7].

Currently, the most common clinical treatment consists of a corneal transplant (keratoplasty) from a human donor. The success rate for this type of allotransplant is about 80% in low-risk scenarios such as non-inflammatory diseases [8]. However, the worldwide demand for donor corneas amply exceeds the available supply. The estimated availability of corneas is 1 per 7 people in developed countries and 1 per 70 worldwide [9,10]. Furthermore, an aging population with a longer life expectancy contributes to poorer quality donations (as the corneas are older) and a greater need for corneas. Additionally, a general rise in refractive surgery is exacerbating this gap, since laser-treated corneas are not considered suitable for transplantation. Moreover, in cases where there has been previous inflammation, a high rate of rejection occurs. Indeed, nine years after engraftment, more than 60% of such transplanted corneas are rejected [11,12]. In cases with multiple failures of previous grafts, new breakthroughs are required to obtain better biointegration of artificial implants into the corneal stroma, as they are no longer suitable for normal corneal transplant or current prostheses, so no solution is available for them. Lamellar keratoplasty (transplantation replacement of only one out of the three layers of the cornea) is partially solving the problem of cornea undersupply. The pooled odds ratio for penetrating keratoplasty rejection over lamellar rejection is 2.02 [13]. Rejection rates long-term (5 years) of lamellar keratoplasties are: DALK ranges between 1.7 and 13%, DSAEK ranges between 5 and 11.4%, and DMEK ranges between 1.7–2.8% [14]. The success rate of DMEK and DSAEK is 90% five years after engraftment [15,16]. However, in high-risk cases, rejection still occurs at a high rate.

**Figure 1:**
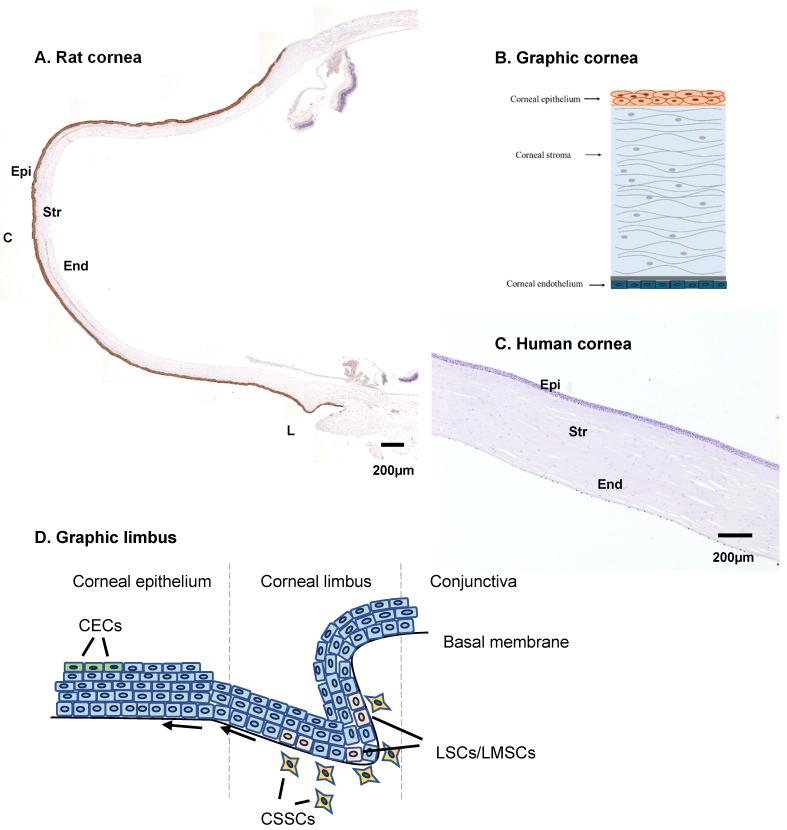
(**A**). Histological section of a rat cornea showing cytokeratin 12 staining along the epithelial layer in brown. L: Limbal area. C: Central cornea. (**B**). Graphic representation of the three cellular layers of the cornea in a histological section. (**C**). Histological section (H&E) of a human cornea showing the three cellular layers of epithelium (Epi), stroma (Str), and endothelium (End). (**D**). Graphic representation of the cellular components of the cornea at the limbal area. CECs: corneal epithelial cells, CSSCs: Corneal stroma stem cells, LMSCs: Limbal mesenchymal stem cells, LSC: Limbal stem cells. Scale bars: 200 µm.

A full cornea consists of three main layers (Figure 1):

The epithelium is the outer layer, formed by corneal epithelial cells (CECs) with high proliferative characteristics. It measures about 50 microns (about 10% of the thickness of the cornea). Epithelial cells are constantly being produced by stem cells of the epithelium located in the limbus and sloughed off in the surface of the eye, so in most cases this layer does not pose a regeneration problem after injury [17].

The stroma is the middle layer and is approximately 500 microns thick (about 90% of the thickness of the cornea). It is formed by corneal stroma cells (keratocytes) and strands of collagen fibrils, which form the structure of the cornea. These fibrils are uniform in size and arranged parallel to the cornea surface in from 200 to 300 flat bundles that extend across the entire cornea. Keratocytes have only limited regeneration properties and only a small amount of damage to this layer can be regenerated naturally.

The endothelium is a monolayer of corneal endothelial cells (CEnCs), measuring about 5 μm (around 1% of the total thickness). CEnCs have no proliferation capacity and only hypertrophy can partially solve a minor injury or degeneration to this layer.

Obviously, cell therapy applied to every one of these layers will expand current therapeutic options, reducing the cost of ophthalmological interventions and increasing the effectiveness of surgery. In this review, we focus on recent advances using mesenchymal stem cells (MSCs) from different sources, aimed at replacing the damaged cells of the three corneal layers.

Before any cell therapy intervention with MSCs, quality controls must be performed. Quality control for MSC-based cell therapy is of crucial importance, as MSCs are donor-derived primary cells, and their batch-to-batch variation is well-documented in numerous studies. The heterogeneity of MSCs is affected by multiple factors, including variations in tissue sources, the intrinsic characteristics of each donor, such as age and lifestyle—remarkably, umbilical cord-derived MSCs (UC-MSCs) exhibit considerable variability—and the processes involved in their isolation, expansion, and administration, all of which can impact their therapeutic efficacy [18]. In fact, we have observed first-hand variability in adipose-derived stem cells (ADSCs)’ ability to differentiate into corneal limbal stem cells (LSCs) depending on the donor’s age and BMI [19]. This variability can be significantly reduced by implementing standardized protocols with reproducible manufacturing processes and stringent quality control measures. These measures typically include cell analysis (e.g., viability, standardized markers, and differentiation capabilities, as already established by the IFATS), safety evaluations (such as procoagulant tissue factor, mycoplasma, endotoxin, and genomic stability testing), and potency assessments, as highlighted in the study [20]. While enzymatic—by collagenase—digestion remains the most common approach for MSC isolation [21], alternative methods, such as using an explant tissue placed in culture media that promotes the release of cellular aggregates [22] have also been described with the aim of ensuring reproducibility while minimizing batch-to-batch variability, thereby maintaining consistent product quality.

## 2. MSCs for the Corneal Epithelium

### 2.1. Preclinical Trials Using MSCs in Different Diseases

#### 2.1.1. Limbal Stem Cell Deficiency (LSCD)

As stated above, corneal transparency and vision acuity are partially maintained by CECs, which are continuously renewed by LSCs [23], so the epithelium itself is usually not a problem if the limbal compartment is not affected. However, limbal stem cell deficiency (LSCD) leads to the destruction or dysfunction of these stem cells, inducing an up-regulation of proinflammatory and pro-angiogenic factors [24]. This causes persistent epithelial defects, which result in corneal scarring, ulceration, neovascularization, conjunctivalization, corneal opacification, and vision loss [25]. If LSCD is partial, an amniotic membrane (AM) is usually used to protect the eye surface while LSCs proliferate and repair the defect. In complete but unilateral LSCD, LSCs from the contralateral eye are used in surgical CLET and SLET types of transplants. In cases of bilateral LSCD, which is the most common, an extraocular autologous source of stem cells is needed to restore the ocular surface to avoid dependence on allogeneic LSCs that are difficult to obtain, isolate, and culture. Scaffolds are often used for the transplantation of MSCs for increased cell adhesion and more pronounced and prolonged improvement. Some of those used are AM [26,27], scaffolds made of fibrinogen [28], fibrin-agarose hydrogel [29], or chitosan hydrogel [30], as shown in Table 1. There are recent reviews that specifically address this issue of the different scaffolds used for corneal regeneration such as Wang et al. [31].

##### Undifferentiated MSCs

The use of ADSCs for the treatment of corneal epithelial damage is a feasible and effective alternative since ADSCs are easily extracted from multiple tissues and are easy to expand. A common way to harvest ADSCs is through liposuction performed voluntarily by patients. The orbital fat of the eye is also a source of ADSCs (OADSCs). It has been shown to reduce macrophage infiltration and induce nitrous oxide synthase (iNOS) production in a mouse model of alkali burn-induced limbal damage, with topical OADSC treatment being more effective than intralesional injection, as it allows for a better cell distribution [55]. Galindo et al. [26] showed that ADSCs seeded on an AM scaffold improved the corneal and limbal epithelial cell phenotype in an LSCD rabbit model, partially recovering the expression of characteristic limbal (cytokeratin-15 (CK15) and p63) and epithelial (CK3 and E-cadherin) markers [26]. However, they could only demonstrate an improvement in the cellular phenotype, since they did not obtain significant results in terms of clinical improvement in the transplanted group compared with the non-transplanted group, probably due to the low number of experimental animals.

MSCs present in the limbal stroma itself are also used as a treatment for LSCD. Corneal stroma stem cells (CSSCs) or limbal mesenchymal stem cells (LMSCs) are normally adjacent to the basal membrane within the limbal stroma. CSSCs can differentiate into keratocytes to repair the damage, expressing aldehyde dehydrogenase 3A1 (ALDH3A1), aquaporin 1 (AQP1), keratocan (KERA), and prostaglandin D2 synthase (PTGDS) marker genes [82]. Both CSSCs cultured in the presence of TNFα and the differentiated keratocytes from CSSCs, embedded in human fibrinogen scaffold, led to an increase in the expression of TNFα-induced protein 6 (TSG-6) in a corneal wound model in mice. This resulted in the downregulation of neutrophil migration, reducing infiltration due to the expression of CD11b+/Ly6G+ neutrophils and myeloperoxidase, thus preventing scarring [28]. TSG-6 secreted by MSCs is critical for the reduction of corneal opacity and inflammation. Other authors have reached the same conclusion with BM-MSCs treatment using rat and mouse models of chemical injury-induced LSCD [83,84].

AM has also been used as a scaffold for the transplantation of LMSCs, either alone [85] or previously co-cultured and transplanted together with human limbal epithelial cells (hLECs) [86]. EGFP-labeled LMSCs were seeded on decellularized AM and the tissue-engineered graft was sutured in rabbits with total LSCD. They partially restored the squamous corneal-like epithelium four weeks after transplantation, reaching 90% restored epithelium at week 12. However, no EGFP-labeled cells were found in the regenerated central epithelium, only at the periphery [85]. These results agree with previous studies showing that LMSCs decrease inflammation and neovascularization in animal models with LSCD induced by alkali burn, showing better results by subconjunctival or topical administration [41]. In a comparative study of LMSCs and LMSCs co-cultured with hLECs (LMSCs-LEC) in rabbits with acute ocular surface wounds, LMSCs-LEC further improved the speed of re-epithelialization reaching a 90% of healing seven weeks after surgery. Although re-epithelialization was successful in both cases, in the LMSCs transplant group the epithelium grown was conjunctival, while in the LMSCs-LEC group, no goblet cells from the conjunctiva were found. However, after 12 weeks, no retained human epithelial cells were detected in the regenerated epithelium [86].

Recently, Long et al. [27] transplanted UC-MSCs inside an AM scaffold, onto which LSCs were seeded into a total LSCD model in rabbits. This tissue-engineered corneal epithelium constructed using UCM-MSCs showed improved transparency results and greater resemblance to the phenotype of natural corneal epithelial tissue, as well as higher levels of cellular progenitor (CK14 and p63) and corneal epithelial (PAX6) gene expression [27].

The benefits of using bone marrow-derived MSCs (BM-MSCs) have been extensively studied. BM-MSCs reduce corneal inflammation, CD4+ cell infiltration, IL-2, and interferon-gamma (IFNγ) expression [87], TFNα production [77,84], and angiogenesis development (through VEGF and MMP2) [77,87]. In addition, they increase the expression of anti-inflammatory cytokines IL10, TGFβ [77,87], IL-6 [84,87], and anti-angiogenic cytokine trombospondine 1 (TSP-1) [77,87]. The first evidence that BM-MSCs could be used for the reconstitution of damaged corneas was found in 2006 when Ma et al. [57] placed AM with BM-MSCs and evaluated their efficacy by comparison with LSCs in rat corneas with chemical burn-induced LSCD, decreasing inflammation and angiogenesis in both cases. Other authors have previously cultured BM-MSCs with cytokines, such as keratinocyte growth factor (KGF-2) and autologous serum, before transplantation with AM in rat models. This resulted in even better results than the use of BM-MSCs seeded on AM, with 90% transparency, 70% lack of neovascularization, and 100% epithelium damage limited to less than 1/4 of the cornea [62]. The use of BM-MSCs in an alkaline burn-induced LSCD model in rabbits showed good histological reconstruction and increased vimentin expression compared to the controls [88]. In this type of alkali injury, the level of ROS production increases, leading to oxidative stress [89]. BM-MSCCs inhibit the ROS-NLRP3-IL-1β signaling pathway [90], restoring the antioxidant/prooxidant balance in the regenerated corneal epithelium [89]. Several treatments have been tested in a rat model of chemical injury-induced LSCD: BM-MSCs, conditioned BM-MSC medium [87], and BM-MSCs in combination with a polysaccharide hydrogel obtained from Hardy Orchid [77]. The latter produced better results with subconjunctival administration in combination with topical administration, significantly decreasing corneal opacity in 7 days. Another type of scaffold used is nanofiber scaffolds, which, when seeded with BM-MSCs and transplanted into alkali injury-induced LSCD rabbit eyes, have shown biocompatibility and regeneration of the epithelium with better results than ADSCs or LSC nanofiber [42].

Dental pulp-derived stem cells (DPSCs) have MSC markers and have been studied in comparison with LSCs as an alternative to LSCD in vitro [91] and in vivo treatment. A sheet of tissue-engineered human immature dental pulp stem cells (hIDPSCs) was transplanted and then covered with AM in a rabbit model of LSCD induced by burn epithelial damage. In only three months, neovascularization was reduced, increasing transparency and achieving a positive effect on the recovery of natural epithelial cell morphology [58,92].

As discussed above, AM is often used as a scaffold to transplant MSCs into affected eyes, but MSCs present in the membrane itself and in the amniotic fluid (AM-MSCs) have also been used. AM-MSCs have been shown to reduce opacification and neovascularization in a rabbit animal model of corneal alkali wounds [93].

In conclusion, the use of scaffolds seeded with MSCs helps to ameliorate epithelial damage when compared to the use of MSCs alone, providing optimal cell support, migration, and proliferation, resulting in better regenerative outcomes. Common use of AM in ophthalmology has shown beneficial results, but the use of natural hydrogels or synthetic nanofibers as scaffolds for ADSCs or BM-MSCs could be a cheaper and more readily available alternative, as biocompatibility and disease improvement have been reported in many of the above studies. However, the ideal scaffold would be collagen-based, replicating corneal composition, as it is the main component [94].

##### Differentiated MSCs

Another alternative to the use of naïve MSCs is the differentiation of these cells into CEC or LSCs, due to the intrinsic potential of MSC to differentiate into multiple cell lineages.

ADSCs differentiated to CECs or cultured in a specific LSC medium for the treatment of LSCD in rat, mouse, or rabbit models [23,24,25,95] have been shown to enhance their regenerative activity, reducing inflammation and restoring corneal transparency. The authors used different ways to differentiate MSCs into CECs and thus achieve re-epithelialization as fast and effectively as possible. Bandeira et al. [25] used a differentiation medium containing an inhibitor of glycogen synthase kinase-3 (GSK-3β) and transforming growth factor-β (TGF-β) signaling, leading to an accumulation of β-catenin, positively regulating long-term self-renewal of neural stem cells [25]. Another approach is to directly transfect the PAX6 gene (an eye development-associated transcription factor required for LSCs and CECs differentiation) [23]. Venugopal et al. [95] used a coating of a thermoresponsive polymer N-isopropylacrylamide-co-glycidylmethacrylate (NGMA) and cultured MSCs in a limbal explant-conditioned medium [95]. Casaroli’s group used a conditioned medium from human CECsor a defined supplemental hormonal epithelial medium (SHEM) containing DMEM/F12, fetal bovine serum (FBS), epidermal growth factor (EGF), insulin-transferrin-sodium selenite, hydrocortisone, triiodothyronine, and adenine to enhance cell proliferation [24]. Sikora et al. [96] differentiated ADSCs into CECs by co-culturing them with LSCs [96] and Nieto-Miguel et al. [97] used basal medium (DMEM with FBS, sodium pyruvate, and L-glutamine) conditioned with CECs or limbal fibroblast. However, these are not meaningful options if the objective is to find an alternative to the use of naïve LSCs or CECs.

Wharton’s jelly, present in the umbilical cord, is a source of MSCs (WHJSCs) capable of in vitro transdifferentiation to CECs expressing CK3/12, plakoglobin (PKG), zonula occludens 1 (ZO1), and connexin 43 (CX43) markers [29,98]. Garzon et al. [29] generated a fibrin-agarose-based hydrogel scaffold containing keratocytes to simulate an artificial stroma. Additionally, they seeded epithelial cells or WHJSCs cultured for seven days with an EGF-enriched medium to differentiate them into epithelial cells. After 28 days, the culture medium of the most superficial part was removed to induce stratification of differentiated epithelial cells in an air–liquid culture [29]. Nguyen et al. [98] used a combination of retinoic acid (regulator of specific genes in cell differentiation), SB-505124 (TGF-β inhibitor), preventing the transition from epithelium to another cell lineage, bone morphogenetic factor 4 (BMP4), and EGF for the first three days of differentiation, followed by supplementing with SHEM for a further six days, generating corneal epithelial-like cells in vitro [98].

BM-MSCs are also often differentiated into CECsfor transplantation. Some authors have differentiated them by transfecting the PAX6 gene, similar to the approach with ADSCs [99], co-cultured with CSSCs [59] or using limbal epithelial cell medium (SHEM medium with cholera toxin to accelerate cell growth) [60], achieving expression of characteristic markers such as integrin β-1, PAX6, ABCG2, CK3/12, and 19 in vivo [59,60,72]. In LSCD animal models, CECs induced from BM-MSC have been transplanted and seeded on AM, showing the ability to remodel the epithelial layers and restore normal morphology with positive immunostaining of CK3 and p63 [59,60] or using fibrin gel to successfully reconstruct the damaged cornea in a rabbit LSCD model [72].

**Figure 2:**
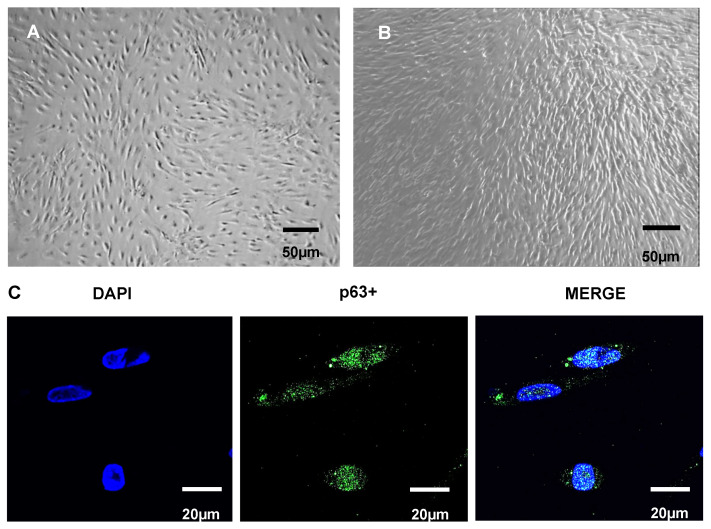
Limbal stem cells (LSCs) differentiated from human adipose-derived stem cells (ADSCs). (**A**). Phase-contrast microphotograph of undifferentiated human ADSCs in vitro. (**B**). Phase-contrast microphotograph of differentiated human LSCs in vitro. (**C**). Confocal microscopy images showing p63+ immunofluorescence of LSCs (green) at day 20 of culture on a vitronectin coating. Nuclear staining in blue. Bars: 50 or 20 µm as indicated.

Obviously, these approaches aim at short-term regeneration of the epithelial layer, as they are not LSCs. Recently, our group has successfully differentiated ADSCs to LSCs in vitro (Figure 2A,B) using FBS, SB-505124, Wnt inhibitor (IWP-2), and FGF-2 for seven days, followed by an LSC induction culture step in modified SHEM, supplemented with pigment epithelium-derived factor and KGF-2 for 10 more days, rendering positive p63 LSCs (Figure 2C) [19].

With regard to the route of administration, systemic administration is not a practical option for the treatment of LSCD in patients from a clinical point of view. Topical administration seems to be more effective in the acute phase and subconjunctival administration is better in later stages of the disease, as the former is sometimes insufficient, although a combination of both may be the best alternative [41,49,77]. More studies are needed to demonstrate the effects of MSCs differentiated into CECs or LSCs without the use of naïve LSCs. Ideally, these MSC-differentiated LSCs or, alternatively, MSC-differentiated CECs should be transplanted directly or using scaffolds for greater cellular activity in the damaged area.

#### 2.1.2. Corneal Ulceration and Keratitis

Corneal ulcer or keratitis is a lesion caused by infections, trauma, or inflammation, among other factors [44]. This disease is common in domestic animals and causes a loss of the epithelium with stromal exposure [45], which can lead to the development of scarring, loss of vision, and even perforations [100]. For the first time in 2019, 26 dogs with corneal ulcers were treated subconjunctivally and topically with allogeneic ADSCs. After 14 days of cell therapy, 84.6% had a significantly lower ulcer depth, demonstrating the safety and effectiveness of ADSCs usage [44]. Although good progress in ulcer reduction was evident, there was no control in this study. Ovarian-derived MSCs (OMSCs) have been shown to express characteristic mesenchymal markers (CD90+, CD44+, CD34− and CD45−) and a wide range of differentiation potential [101]. In a preclinical study, a dog with a corneal ulceration-derived descemetocele (deep corneal ulcer characterized by thinning of the epithelium and stroma reaching the Descemet membrane) was treated with OMSCs. The cells were injected into the conjunctiva with an additional topical application. The improvement was remarkable by day 3, as the size of the injury decreased. After 30 days, the dog’s descemetocele had healed completely, along with the absence of edema and hyperemia, and the corneal opacity finally disappeared by day 75 [45]. This was a preclinical study showing the efficacy and safety of MSCs in ulcer healing, but only one animal was treated, there were no controls, and the source of MSCs was not common or easy to obtain. ADSCs may be the most effective and widely available option in terms of ulcer reduction, although further studies with more experimental animals and controls are needed.

In a mouse model with cryoinjury-induced epithelial damage, AM-MSCs from amniotic fluid reduced keratitis and promoted epithelial cell growth, eliminating the necrotic structure and restoring normal morphology. They also increased levels of cell proliferation marker ki67, binding protein ZO1, and apoptosis inhibitor Bcl-2 expression, with lower levels of leukocyte-activator interleukin 1 beta (IL-1β) [102].

#### 2.1.3. Dry Eye Disease (DED)

Dry eye disease (DED) is one of the most common pathological corneal conditions. DED is caused by multiple factors. Its most characteristic effects are inflammation of the ocular surface, alteration to functionality, and homeostasis of the lacrimal gland and tear film. All this causes pain, a significant decrease in visual acuity [103] and epithelial dysfunction in patients [104].

BM-MSCs have been tested for the treatment of DED in mouse and rat models administered as drops [105], subconjunctivally [48], and intraperitoneally [51]. Inflammatory DED can be induced in animal models by using benzalkonium chloride (BAC) administered as drops. Labeled BM-MSCs administered as drops in a BAC-induced DED rat model decreased leukocyte infiltration after seven days, showing migration into the conjunctival epithelium and meibomian glands, which prevented the evaporation of tears [105]. The limitation of this study is that it did not recreate a complete DED model, only an inflammatory type of DED. DED is frequently associated with other diseases, such as graft-versus-host disease (GVHD), as some of its side effects are inflammation, destruction of the ocular surface, vision loss [48], and Sjögren’s syndrome (SS), a chronic inflammatory autoimmune disease that affects the exocrine glands, such as the salivary and lacrimal glands [51]. In all these types of cases, the use of BM-MSCs has been shown to decrease the inflammation associated with the disease. Indeed, when analyzing the tear glands after four weeks of treatment in a mouse SS-DED model, the size of the lymphocytic foci decreased by 40.5%. Tear production also increased in the damaged eyes, raising the expression of water channel aquaporin 5 mRNA, thus improving the secretion and functionality of the lacrimal gland [51].

Dietrich et al. [103] used experimental aqueous-deficient dry eye (ADDE, a type of dry eye disease characterized by a reduction of lacrimal secretion commonly caused by SS) in mouse models to transplant EGFP-labeled lacrimal gland-derived MSCs (LG-MSCs) directly into the affected lacrimal glands. These LG-MSCs were still detectable 21 days after transplantation, in the early phase of tissue repair, decreasing TNF-α expression and increasing immunoregulatory factor IL-6. In addition, 62% of the total LG tissue recovered acinar architecture, 25% more than in the control group, where spontaneous recovery occurred after saline injection [103]. The benefits of the use of LG-MSCs have been reported, but with clinical insight, this source of MSCs is not as available as BM-MSCs.

### 2.2. Preclinical Trials of Cell-Free Therapy with MSCs-Derived Extracellular Vesicles (EV-MSCs) in Different Diseases

Signaling molecules, proteins, lipids, and nucleic acids are naturally released from cells into the extracellular space due to an invagination of the cell membrane, forming the so-called extracellular vesicles (EVs), surrounded by a phospholipid bilayer. All cell types secrete EVs, with content differing depending on the cell of origin and its function. They release bioactive molecules to other cells or interact with surface membrane receptors, inhibiting or activating different signaling pathways [32,106]. EVs suppress the activation of immune cells and the expression of proinflammatory factors and fibrotic genes, blocking neutrophil infiltration and leading to the restoration of normal tissue morphology [34]. Exosomes are a type of EVs, characterized by their nanoparticle size, ranging between 30–150 nm, which have been identified as important mediators of therapeutic functions via cell communication [107]. MSC-derived EVs are considered important paracrine mediators through transferring microRNAs [39,108]. Both EVs and miRNAs are required for wound closure [34,108], functioning as therapeutic nanoparticles [107]. Multiple miRNAs, capable of regulating gene expression, are naturally present in exosomes, but a desired chemically synthesized miRNA can also be encapsulated in exosomes.

EVs secreted by MSCs derived from the lacrimal gland [109], umbilical cord [108], bone marrow [32,40,110], adipose tissue [36,37,39,111], human placenta [35], corneal stroma [33,34], and even induced pluripotent stem cell-derived mesenchymal stem cells (IPSC-MSCs) [30,32,107] have been reported to accelerate corneal wound healing in animal corneal epithelial damage models.

#### 2.2.1. EV-MSCs for LSCD

EVs from human placenta-derived MSCs (hP-MSCs) ameliorated alkali injury of the cornea in a mouse model, inhibiting angiogenesis and inflammation. EV-hP-MSCs stimulated the proliferation and migration of CECs, resulting in a phenotypic recovery of the cells of the epithelial layers, restoring their natural shape [35]. Exosomes from MSCs extracted from human corneas (CSSCs) were administered topically in a mouse corneal wound model, resulting in 77% corneal wound healing after 72 h compared to 42% in the control group [33]. In a preclinical comparative study between BM-MSCs and exosomes derived from labeled BM-MSCs, both were administered subconjunctivally in a corneal epithelium-deficient diabetic mouse model. After 24 h, 48 h, and 72 h, the corneal tissues were analyzed, and a similar epithelium healing rate was found. The distribution of exosomes was confirmed in the limbal epithelium, but not in the wound [40]. From these studies, we may conclude that the method of application of EVsalso affects their effectiveness, with topical application appearing to be more effective than subconjunctival application.

In a rabbit model of alkali burn-induced LSCD, a thermosensitive diethylene glycol monomethyl ether methacrylate (DEGMA)-modified hyaluronic acid hydrogel (THH) was used as a scaffold promoting a controlled release system of exosomes derived from ADSCs transfected with miRNA-24-3p by subconjunctival injection. In the THH/Exos-miRNA 24-3p group, exosomes promoted the migration of CECs, as demonstrated by the higher expression of MMP9 and EGFR compared to the control group, achieving complete corneal epithelial wound healing already at day 3 only in the exosome-treated group [39]. THH use did not decrease the number of goblet cells in the epithelial layer by itself, but there was no rejection. Other authors, such as Tang et al. [30], agree that topical administration with a scaffold is the most effective approach [30]. In their study, thermosensitive chitosan hydrogel was used as a scaffold for IPSCs-MSC-secreted exosomes containing miRNA-432-5p. Scar formation was prevented by suppressing TRAM2, a regulator of collagen production in CSSCs [30].

Other authors, such as An et al. [110], showed that using BM-MSC-conditioned medium after 72 h of culture accelerated wound closure significantly compared to that collected after 48 h in a mouse model of corneal epithelial wound healing. When diluting the conditioned BM-MSC medium to half the concentration, they obtained worse results than with the original concentration, although they still obtained better results than in the control group [110]. However, because they used a BM-MSC-conditioned medium, which is influenced by paracrine growth factors secreted by the cells themselves in addition to exosomes, the results may not be comparable. More preclinical trials with longer treatment times are needed, as well as establishing the optimal concentration of exosomes, optimization of the cell source, and standardization of culture conditions to allow for comparisons between studies.

#### 2.2.2. EV-MSCs for DED

EVs extracted from ADSCs administered as drops in a DED mouse model reduced expression levels of inflammatory factors such as IL-1β, IL-6, IL-1α, IFNγ, and TNF-α, as well as inactivating NLRP3 and caspase-1 [36,37]. With the same topical application method, BM-MSCs exosomes have also been shown to increase tear secretion and re-epithelialization in a GVHD-DED mouse model [38]. The efficacy of EV-BM-MSCs or EV-IPSC-MSCs in inhibiting lymphocytic infiltration and preventing salivary and lacrimal gland inflammation was shown in a model of non-obese diabetic mice with SS-DED, obtaining similar results to the use of BM-MSCs or IPSC-MSCs [32].

There is no consensus on the use of EVs as being more effective than the use of MSCs themselves. In addition, the use of MSC-derived EVs in early or late passages also influences the outcome in terms of immunoregulatory cytokines and miRNA content [112]. Topical administration is most commonly used, as its application is simpler and thus does not entail the risk of damaging the conjunctiva. As miRNAs have been shown to be essential for ocular wound healing, DED could benefit from more studies including miRNAs to improve the statistics and further elucidate the advantages of the use of EVs versus MSCs.

### 2.3. Clinical Trials Using MSCs in Different Diseases

Regulatory hurdles remain a significant bottleneck in advancing allogenic MSC therapy, which may be one reason why corneal cell therapy has not progressed as expected. In Europe, MSCs are considered to be advanced therapy medicinal products (ATMP), governed by a specific ATMP regulatory framework, the Regulation 1394/2007/EC and Directive 2009/120/EC [113]. The development of ATMPs is also regulated by the guidelines on good manufacturing practice (EU GMP-ATMPS), which involves applying many requirements to ensure their safety, quality, and efficacy. These requirements have a high manufacturing cost, as they require highly specialized human resources with GMP manufacturing experience and special laboratory conditions. In addition, it is necessary to provide pre-clinical studies on the proposed medicine product, and GMP implementation has to be considered during these studies to avoid being trapped in the translation process [114,115].

In the United States, MSCs are legislated by 21 CFR 1271 as human cells, tissues, and cellular and tissue-based products (HCT/P) and, as in Europe, they are conducted in accordance with the good manufacturing practice (21 CFR 211). In contrast to Europe, the requirements in the United States are less comprehensive. Restrictions are limited to federally funded research; there are no limitations on research when funding comes from private investors [116]. For manufacturing, the presence of a qualified person is not required, and early phases I or II trials are not subjected to regulatory inspection [115].

In addition to the problem of variability explained above regarding the quality control for MSC-based cell therapy, the lack of consensus on naming and distinguishing some MSCs from different tissues and the absence of agreement on the optimal culture conditions at the clinical scale between different MSC manufacturing centers creates a major difficulty when legislating and complicates the comparison of results in terms of safety and efficiency across clinical studies [115,117]. Another problem is the inability to ensure that the product is sterilized because of the incapacity to do terminal filtration [118].

**Figure 3:**
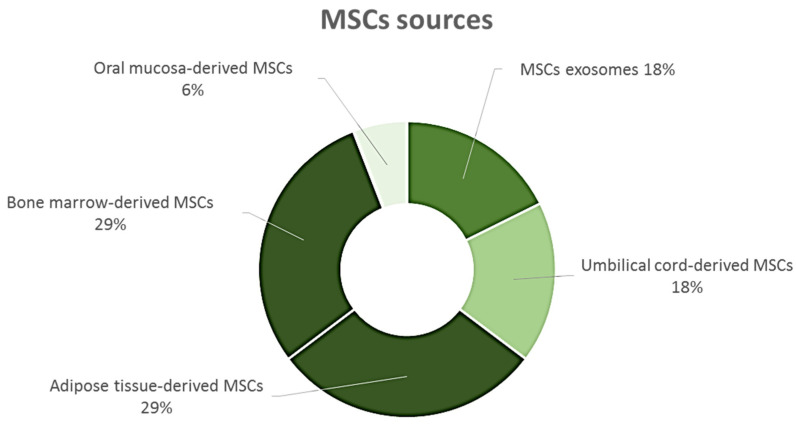
Mesenchymal stem cells (MSCs) sources to treat corneal epithelial diseases in 18 clinical trials published in ClinicalTrials.gov. Available online: https://clinicaltrials.gov/ (accessed on 24 October 2024) at the time of submission.

These regulatory hurdles explain the lack of advanced phase III trials and why most MSC-based trials are still in the early I or II phases and currently only approximately 1% of MSC-based cell therapy products have been approved for market commercialization worldwide [114,115]. With respect to corneal epithelium, currently 18 clinical trials have been registered in the U.S. National Library of Medicine (ClinicalTrials.gov. Available online: https://clinicaltrials.gov/ (accessed on 24 October 2024)), in which patients with corneal epithelial diseases have been treated with MSCs either directly or indirectly (such as the use of EVs derived from them). There are a number of clinical studies (ongoing, active, recruiting, completed, or of unknown status) using MSCs from different sources (bone marrow, umbilical cord, adipose tissue, and oral mucosa, Figure 3) applied in different ways, either with eye drops, subconjunctival injections, graft transplantation with AM, or intravenous injection.

Three clinical trials have been published on the treatment of LSCD (NCT01562002, NCT03226015, and NCT01808378), six on treating DED (NCT05738629, NCT04213248, NCT03878628, NCT04615455, NCT02291770, and NCT05784519), two on superficial ocular burns (NCT02325843 and NCT03237442), one on cicatricial conjunctivitis (NCT05520086), and NCT05200000 on the treatment of chronic keratitis. There are five on general epithelial diseases: NCT05705024 is on patients suffering from persistent corneal epithelial defects (PCED), corneal ulcer or neurotrophic keratopathy; NCT04484402 is on inflammatory-dystrophic diseases of the cornea such as ulcerative, neurotrophic or contusion keratitis, and keratopathy and burns of the cornea; NCT0462626583 is on PCED, corneal ulcer, neurotrophic keratitis, LSCD, or inflammatory DED; NCT05204329 is on chronic corneal epithelial disease; and NCT05727878 is on participants diagnosed with PCED. Of all these clinical trials, only five have published their results in scientific articles, as shown in Table 2.

#### 2.3.1. Limbal Stem Cell Deficiency (LSCD)

MSC transplantation is established as a treatment option for LSCD because it has been shown to be as safe and effective as CLET for the treatment of LSCD [61]. The clinical study NCT01562002 (Table 2) was the first study to show the benefits of using isolated BM-MSCs to treat LSCD, compared to the usual CLET, a valid therapy for ocular surface insufficiency [121]. In 2019, Calonge et al. [61] showed the results of this study in 27 patients during a 12-month follow-up. They used allogeneic BM-MSC transplantation in 17 patients with an AMseeded with 2.5 × 10^5^ cells for comparison with another group of 11 patients undergoing CLET. A major improvement in the phenotype of epithelial cells in the MSCs transplantation group was seen, with 85.7% success versus 77.8% in the CLET group. None of the cases of patients with non-inflammatory prognoses failed, although it should be noted that in cases where patients had inflammatory diseases, there were problems of efficacy; 57.1% of failures were chemical injuries and 42.9% were immune-mediated inflammatory diseases [61].

A recent report presented the results of a clinical trial using autologous ADSCs in eight patients with bilateral LSCD (NCT01808378, Table 2). The patients were injected with 4 × 10^5^/0.4 mL of autologous ADSCs into each limbal conjunctival quadrant, and a further 4 × 10^5^/0.4 mL of autologous ADSCs was administered in drops. After 20 min, the cornea was covered with AM. After 86.5 months of follow-up, none of the LSCD patients showed epithelial defects [50]. As limitations to the study, the distribution of the cells was not monitored and, as administration was subconjunctival, there is a possibility the cells could have been distributed into the bloodstream. Not all patients had severe LSCD, so recovery may in part be due to natural re-epithelialization with residual LSCs [50]. Nevertheless, the most significant limitation of this clinical trial is the small number of patients and the lack of a control group.

#### 2.3.2. Dry Eye Disease (DED)

The use of MSCs for the treatment of DED was shown to be safe and effective for the first time in a clinical study by Weng et al. [46] (Table 2). They used intravenous injection of allogeneic ADSCs for the treatment of DED associated with GVHD in 22 patients. The enrolled patients received 2 × 10^6^/kg of ADSCs, six doses in total: during the first two weeks of treatment the dose was twice a week and for the following two weeks it was only once a week. After a 3-month follow-up, improvements were achieved in dry eye scores, ocular surface disease index scores, Schirmer’s test results, and overall clinical symptoms in 54.55% of cases. Regarding the immune system, in the MSC treatment group, there was a significant increase in the presence of CD8 + CD28-immunoregulatory cells, which was not seen in the control group. In addition, higher levels of IFNγ and IL-2, Th1, and Th2 cytokines were detected. The total dose administered was not directly related to improvements in symptoms, so the optimal dose for clinical improvement in most patients was not identified, but the safety of allogeneic ADSCs treatment, even by intravenous injection, in patients was reported [46].

In the study by Møller-Hansen et al. [119] (NCT03878628, Table 2) for the treatment of ADDE secondary to SS, 11 × 10^6^/0.5 mL allogeneic ADSCs was injected transconjunctivally into the lacrimal gland (LG) in one eye. Seven patients were followed for four months, with improvements to parameters such as increased tear film breakup time and ocular surface disease index, among others. However, these improvements were also detected in the untreated eye, diminishing the conclusions regarding efficacy from the study. In a more recent clinical trial from the same group, Møller-Hansen et al. [119] (NCT04615455, Table 2) presented their results after patients received a single injection of 22 × 10^6^/1 mL ADSCs (double the dose of the previous study), or vehicle into the LG of one eye. This trial also involved a higher number of patients (54) and a longer follow-up time (12 months) in the same type of patient (ADDE-SS), although only patients with a severe diagnosis were included. Tear film stability and tear production only increased significantly in the ADSCs treatment group. However, in the case of subjective symptoms of DED, parameters were reduced by 40% in the ADSCs and vehicle group compared to the control group without treatment, probably because patients in both experimental groups (ADSCs and vehicle) were prescribed the use of lubricating eye drops, and the results could have been affected because they were unable to quantify their proper use [120].

Another clinical trial was carried out by Zhou et al. [38] (NCT04213248, Table 2), in which exosomes derived from umbilical cord-derived mesenchymal stem cells (exo-UC-MSCs) were used to treat GVHD-DED. Participants were administered 10 µg/50 µL exo-UC-MSC in drops per eye for two weeks, four times a day, in a group of 14 patients (28 eyes) during a three-month follow-up. Treatment with exo-UC-MSCs resulted in corneal epithelial regeneration and decreased inflammation. In the cornea and conjunctiva, they observed a significant decrease in the expression of IL-17a, IL-6, IL-1B, and CD86, proinflammatory genes, and an increase in anti-inflammatory macrophage markers CD11b+, CD11c−, and CD206+. In addition, in the treatment group, there was an improvement in the study parameters, such as an increase in tear film breakup time and tear secretion, and a lower rate of ocular surface disease [38]. This form of administration by drops is a less invasive treatment option than in other clinical trials using injections.

Although it is difficult to compare the studies and draw conclusions, as they involved patients with DED caused by different types of diseases (GVHD and ADDE-SS) and used different inclusion criteria, all the clinical trials have shown that the use of MSCs is safe and effective for DED treatment, mainly due to its immunomodulatory activity. Also, the use of exo-MSCs is promising in corneal regeneration, however, further research is needed [122].

## 3. MSCs for the Corneal Stroma

The corneal stroma is the middle layer of the cornea, composed of three regions: Bowman’s layer, the stroma proper, and Descemet’s membrane. The stroma proper is a layer predominantly composed of type I collagen, water, extracellular matrix molecules, and keratocytes. It is essential for transparency, avascularity, mechanical properties, and maintaining the overall shape of the cornea [123]. The stroma proper has a well-organized architecture of an extracellular matrix (ECM) composed mainly of collagen fibrils arranged in orthogonal lamellae, which are essential to corneal functionality and shape retention [124,125]. Corneal keratocytes, originating from neural crest cells, are responsible for depositing the ECM during late embryonic development. These quiescent cells, situated between stromal lamellae, synthesize collagen and proteoglycans, notably lumican and keratocan, to maintain corneal transparency [123]. Upon injury, the keratocytes are activated, resulting in either cell death or repair, depending on the severity of the damage [126], and they transdifferentiate into fibroblasts and myofibroblasts [127]. Myofibroblasts, characterized by the presence of alpha-smooth muscle actin, can proliferate excessively and contribute to the formation of corneal scarring and fibrosis [128]. CSSCs are mainly located in the anterior stroma, near the corneal epithelium. Their location enables a rapid response to corneal injuries and contributes to maintaining corneal immune privilege. In the limbal stroma, there are more CSSCs with a quiescent condition and regenerative capacity [129]. CSSCs present genes characteristic of early corneal development, PAX6 and SIX2, in addition to genes characteristic of mesenchymal stem cells, such as ABCG2, BMi1, CD166, cKIT, and Notch1. During differentiation, CSSCs show an elevated expression of keratocyte-associated genes, including keratocan [130].

### 3.1. Preclinical Trials with MSCs in Different Diseases

#### 3.1.1. Corneal Scarring and Opacities

Corneal opacity, characterized by a loss of transparency essential for vision, impairs the ability of the cornea to effectively transmit and focus light. This condition, often caused by trauma, infections, or autoimmune responses, frequently results in scarring and is recognized as the fourth leading cause (5.1%) of blindness worldwide. It involves disruption to the function of native corneal stromal keratocytes (CSKs), reducing their production of the proteins necessary for clarity [131].

CSSCs, which can be harvested from clinical biopsies, have the capacity to proliferate through numerous cycles of replication in vitro while maintaining their differentiation potential. Du et al. [132] and Basu et al. [82] found that expanded CSSCs can regenerate normal stromal tissue in a mouse model of corneal scarring, produce transparent ECM, inhibit fibrotic scar formation, thereby promoting native stromal tissue regeneration, probably through a paracrine mechanism, and restore corneal transparency. These findings suggest a promising approach for treating corneal wounds and scars with autologous, xenobiotic-free cell therapies whose immunomodulatory properties make them suitable for treating corneal scarring in humans [130].

The combination of stromal quiescent human corneal keratocytes (qCSKs) with a high-quality intra-stromal delivery in a murine model resulted in the most significant reduction in haze density and area [127]. By contrast, suboptimal cellular delivery often led to a greater and less uniform volume expansion due to leakage or backflow of the injection medium containing qCSKs, which appeared to correlate with more opacity in the eyes of the rats. This experiment underscored the significance of spatial distribution in influencing the therapeutic properties of qCSK injections, as well as activated corneal stromal keratocyte (aCSK), and, to a lesser degree, corneal stromal fibroblast (SF) injections [127]. Today, the use of adult CSSCs is more popular due to their capacity to regenerate corneal structure and transparency. The increase in fibrogenic factors TFG-β1 and TFG-β2 post-injection may make CSSCs from peripheral corneal and limbus less suitable. By contrast, CSKs have a lower tendency to transform into fibroblasts but possess extracellular matrix reorganization capabilities [127]. For stromal regeneration, qCSK therapy is a good option. An article by Jhanji et al. [133] highlights a combinatorial cell therapy approach using CSSCs followed by qCSK. CSSCs can help reduce inflammation and fibrosis, while qCSK can regenerate the native stromal ECM.

Additionally, the corneal stroma has been identified as a source of human mesenchymal stem cells (hMSCs), which express key markers, such as CD73, CD90, and CD105. These cells have the potential for therapeutic applications, but further studies are needed to fully understand their role in corneal repair [134].

There are a number of reviews exploring the status of MSC use for restoring the corneal stroma in different diseases [135,136]. In addition to differentiating into corneal cells, MSC treatments have been shown to be beneficial in reducing inflammation mediators such as IL12, IL6R, and TNF-α in stromal wound mouse models [137].

ADSCs have been studied for topical application to repair lesions produced on the epithelial and stromal layers by laser-induced photorefractive keratectomy (PRK). Over a period of three (16 animals) and seven days (17 animals), mouse eyes treated with ADSCs showed a lower presence of myofibroblasts (α-SMA+ cells), along with improved corneal clarity and decreased haze. By contrast, the control and serum-treated groups showed poorer outcomes [43].

Alkali burns to the cornea often result in significant and irreversible visual disability. Typical clinical complications are corneal ulcers, intense stromal inflammation, and new blood vessel growth in the cornea. Much of this damage is linked to the loss of epithelial limbal stem cells, as extensively explained in the previous section. However, deep alkali burns often compromise the corneal stroma as well. A pioneering study conducted two decades ago yielded promising results in this area, showing that BM-MSCs promote corneal wound healing after alkali burns, either independently or in combination with hematopoietic stem cells [138].

Mucopolysaccharidoses (MPSs) are a group of genetic disorders caused by mutations in lysosomal exoglycosidases, resulting in abnormal accumulation of glycosaminoglycans (GAGs). MPS type VII, due to a mutation in the enzyme β-glucuronidase, is characterized by clinical manifestations, including corneal clouding. In a study involving the transplantation of human UC-MSCs into the intrastromal region of MPS type VII mouse corneas, a reduction in corneal haze was observed one to three months post-transplantation. The results demonstrated that corneas treated in the first and second months showed measurably better integrity compared to those treated in the third month, suggesting that an early intervention may help prevent corneal clouding [54].

##### Differentiated MSCs

SCs offer significant potential for cell therapy due to their ease of extraction. DPSCs do not trigger an allogeneic immune response, as they are non-immunogenic and possess strong immunosuppressive characteristics [139]. Human periodontal ligament (PDL) containing neural crest-derived stem cells could differentiate into a CSK phenotype. Human periodontal ligament stem cells (PDLSCs) were cultured on patterned silk membranes with keratocyte differentiation medium (KDM), consisting of DMEM supplemented with ascorbic acid, bFGF-2, TGF-β3, and the neuropeptide substance P (SP) to investigate its role in promoting keratocyte differentiation over 18 days. The keratocytes expressed collagen types I and V, as well as the corneal stromal proteoglycans lumican and keratocan [70]. In another study, five randomly chosen primary PDL cultures showed comparable efficiency in generating cells expressing CSK markers, such as CD34, ALDH3A1, keratocan, and lumican. To induce differentiation, the same components as the previous group were used, except for DMEM/F12 supplemented with non-essential amino acids. Inserting PDL spheroids into porcine corneas at the stroma led to the migration of PDL-derived CSK-like cells into the host stroma after 14 days. These cells displayed a quiescent state, closely mimicking the characteristics of mature CSKs within the native stroma [140]. MSCs have been shown to spontaneously adopt a keratocyte phenotype in vivo [53,141,142], thus making induction into keratocytes unnecessary. However, in an environment with high inflammation and stromal fibrosis, it is unclear whether MSCs can differentiate into CSKs spontaneously in vivo. In such cases, to enhance efficiency, ADSCs may be differentiated in vitro [143].

#### 3.1.2. Corneal Stromal Thinning

Research has shown that UC-MSC transplants can restore corneal transparency in genetically modified mouse models with congenital defects, such as lumican-null [53] and collagen V-null mice [74]. These UC-MSCs differentiate into keratocytes on the designated area, improving corneal thickness and transparency [53]. UC-MSCs and umbilical cord-derived hematopoietic stem cells (UC-HSCs) were successfully transplanted into lumican-null mice displaying thin, hazy corneas. The UC-MSCs instrastromal transplantation significantly improved corneal transparency and increased stromal thickness in lumican−/− mice, whereas UC-HSCs did not have the same effect due to a significant immune reaction leading to cell loss [74]. The collagen lamellae in the corneal stroma were rearranged in the UC-MSC cases. The cells survived in the mouse corneal stroma for over three months with minimal graft rejection and adopted a keratocyte-like phenotype [74]. UC-MSCs reduced corneal opacity in Col5a1 KO mice, with improvement seen at 14 days post-transplant compared to 7 days in Col5a1 WT mice. By contrast, UC-MSCs are not effective if administered after a delay in treating a keratectomy wound. After 14 days of wound healing, UC-MSCs were applied to the mouse eye surface using a fibrin carrier, but the cells were unable to penetrate the corneal stroma, and the opacity remained uncorrected [74].

Similarly, a 2012 study [142] used BM-MSC, which differentiated into keratocytes when transplanted into the corneal stroma, showing improvements in corneal thickness and transparency. Allogeneic BM-MSCs transplanted into the cornea can survive for up to six weeks without inciting inflammation or an immune rejection response in keratocan-null and lumican-null mouse models [142].

**Figure 4:**
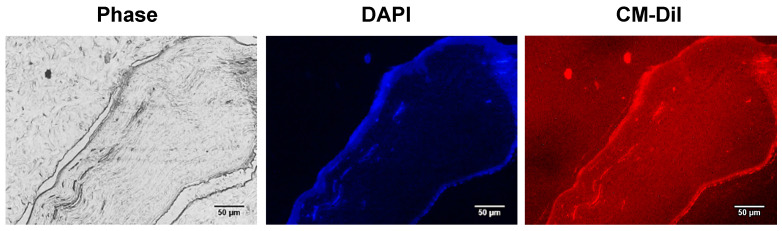
Phase and fluorescent images of rabbit corneas transplanted with CM-DiI-labeled human adipose-derived stem cells (ADSCs) (red). DAPI nuclear staining in blue. Scale bars: 50 µm.

Our group was the first to transplant ADSCs (Figure 4) in the injured corneal stroma of rabbits partially ablated by femtosecond laser. The ADSCs dispersed within the collagen lamina and differentiated into keratocytes, acquiring their dendritic morphology and producing collagens I and VI, usually found in the corneal stroma, but not other abnormal collagens. They also expressed corneal-specific proteoglycan keratocan and ALDH3. Moderate haze was noticed in some of the corneas injected with ADSCs, but no inflammatory reaction or immune rejection was observed [141]. In a later study, decellularized human corneal stromal lamellae biointegrated well, with or without recellularization with human ADSCs [66]. The h-ADSCs differentiated into keratocytes in the grafts. These results prompted the research group to perform a recellularized sheet treatment with ADSCs in a clinical trial (NCT02932852) described below [144].

Our group also showed that h-ADSCs transplanted together with poly(ethyl acrylate) (PEA) membranes can survive for at least three months in vivo and are a promising material for keratoprostheses, improving their clinical potential by optimizing their hydrophilic properties. As the hydrophobicity of PEA increases tissue friction and the risk of ulceration, this led to its modification with hydrophilic units, such as HEA (hydroxyl groups) and AAc/MAAc (carboxylic groups). Low hydrophobicity (10%) improved biointegration and cell adhesion, while high hydrophobicity (20%) reduced cell colonization [80].

### 3.2. Clinical Trials Using MSCs in Different Diseases

The U.S. National Library of Medicine website (ClinicalTrials.gov. Available online: https://clinicaltrials.gov/ (accessed on 24 October 2024)), has five clinical trials involving the treatment of corneal stromal pathologies with MSCs, three related to the study of corneal burn and opacity (NCT03237442, NCT02948023 and NCT03295292) and two (NCT02932852 and NCT05279157) related to keratoconus. However, results are only available for NCT02932852 and NCT03295292 (Table 3).

#### 3.2.1. Corneal Scarring and Opacities

To address corneal stromal scarring leading to corneal haze, a pilot study (NCT03295292, Table 3) was conducted to assess the safety and effectiveness of ex vivo cultivated limbal stromal stem cells (LSSCs) in 15 patients, following phototherapeutic/refractive keratectomy (PTK/PRK) and collagen cross-linking (CXL). Results from this clinical trial two years later showed 13.3% of the LSSC group needed a second operation while the percentage in the control group rose to 60%. These percentages indicate promising outcomes by improving transparency and reducing corneal vascularization in the patients [145]. With the aim of finding a treatment for corneal scars, the same researchers conducted another clinical trial (NCT02948023, Table 3) with 100 patients suffering from corneal scars, ulcers, and burns, also treated with LSSCs. In this case, no results have yet been published.

A double-blind clinical trial in China was performed with 100 patients to evaluate the efficacy and safety of UC-MSCs in the treatment of corneal burns in humans (NCT03237442, Table 3), although no results have yet been published.

#### 3.2.2. Keratoconus

Keratoconus is a corneal atrophy and protrusion disorder characterized by progressive thinning and weakening of the corneal stroma, resulting in progressive astigmatism and vision loss. The disease is marked by the loss of keratocytes and the breakdown of collagen fibers by matrix metalloproteinases. Although current treatments, such as corneal collagen cross-linking and keratoplasty, are frequently performed, they come with limitations and potential risks. To overcome these challenges, researchers are exploring alternative therapies to treat keratoconus, with a particular focus on cell-based approaches [126].

In a pioneering clinical trial by our group (NCT02932852, Table 3 and Table 4), injections of autologous ADSCs into corneal stroma already showed improved corneal transparency, central corneal thickness, and visual function in patients with advanced keratoconus at three months. There were minimal complications, and new stromal collagen production was observed [146]. This study thus demonstrates that stromal enhancement by MSC therapy could be effective for the treatment of advanced keratoconic eyes. In the same clinical trial, decellularized corneal stromal lamina with or without autologous ADSCs transplanted into 14 patients with advanced keratoconus produced full recovery of corneal transparency within three months post-surgery. The advantage of recellularizing the stromal lamina was called into question since there were no notable differences in visual parameters or keratometric measurements, but this could be due to the low number of patients in this pilot study. After one year, groups receiving the lamina showed improved refractive thickness and patients with recellularized laminas had higher cell density, as observed via confocal microscopy [67]. In addition, changes in corneal stroma cell density were evaluated using in vivo confocal microscopy (IVCM) at one year, and a gradual and significant increase in cell density was observed across all three groups. In the group receiving only ADSCs, the increase was more pronounced in the mid-stromal layer, whereas in the groups treated with stromal lamina, the cell density was notably higher on the anterior surface, within the lamina, and on the posterior surface. The best outcomes were in the group receiving recellularized lamina with ADSCs [6].

A study was conducted to evaluate immune cell (IC) infiltration at 1, 3, 6, and 12 months using IVCM. Low numbers of immune cells were observed in all groups, with corneal stromal infiltration ranging from 1.9% to 6.62%, regardless of the time elapsed. However, this IC infiltration was at the non-clinical level [147]. A three-year follow-up indicated moderate improvements across all groups [144]. In conclusion, MSCs, and particularly ADSCs, show great potential for treating corneal stromal diseases [148,149].

A larger study (NCT05279157) with the same treatments is being conducted by the group, but no results are available yet.

Nevertheless, the low number of clinical trials on the treatment of corneal stromal pathologies prevents us from reaching a conclusion, underlining the need for further research to achieve corneal stromal regeneration.

## 4. MSCs for the Corneal Endothelium

The human corneal endothelium is the innermost layer of the cornea, composed of a monolayer of CEnCs derived from the neural crest [150]. They are characterized by the expression of Na^+^/K^+^ ATPase and aquaporin 1 (AQP1) proteins, responsible for the maintenance of corneal hydration homeostasis, zona occludens 1 (ZO1), and N-cadherin (CDH2) proteins, responsible for maintaining the intercellular junctions and their hexagonal shape, and collagen type VIII, a structural component of the basement membrane of the corneal endothelium, Descemet’s membrane [151,152,153,154,155]. Human corneal endothelial cells (hCEnCs) have hardly any regenerative capacity in vivo and the density of hCEnCs reduces with age. When the cell density drops below 500 cells per mm^2^, due to intraocular surgery, trauma, or diseases such as bullous keratopathy or Fuchs’ endothelial dystrophy, this leads to corneal endothelial dysfunction that encompasses corneal hydration, homeostasis deterioration, and, consequently, corneal opacity [156].

Two types of transplants are currently used for endothelial dysfunction: Descemet’s stripping automated endothelial keratoplasty (DSAEK) and Descemet’s membrane endothelial keratoplasty (DMEK) [157,158]. Both techniques have proven their efficacy in more than 15 years of experience and have a high success rate [15,159]. However, these procedures require a donor cornea and worldwide demand for donor corneas currently exceeds the available supply. This global shortage of donor corneas is prompting researchers to look for an alternative treatment, such as stem cell-based therapy.

Several authors have suggested the presence of endothelial progenitors in a niche adjacent to the peripheral endothelium, termed the transition zone [160,161]. This transition zone contains cells expressing stem cell markers (Nestin, OCT3/4, Sox2, and telomerase). Isolated porcine [161] and human [162] transition zone cells were able to generate a monolayer of CEnCs in vitro with characteristic expression of Na^+^/K^+^ ATPase and ZO1. Although further studies on the endothelial progenitor niche are required, their propagation from donor corneas could be a new cell source to generate corneal endothelium for regenerative medicine without compromising the donor tissue limitation, as the peripheral endothelium and the transition zone, where endothelial progenitors are located, are often discarded in endothelial keratoplasties [163]. So far, limited information and only in vitro applications have been explored.

### 4.1. Undifferentiated MSCs

Liu et al. [78] demonstrated that autologous rabbit BM-MSCs implanted on glutaraldehyde-fixed gelatin membranes in a rabbit model of corneal endothelial dysfunction were able to repopulate the corneal endothelium and differentiate into cells with a morphology similar to CEnCs, reduce edema, and recover transparency two months after transplantation. However, Shao et al. [164], who injected human UC-MSCs into the anterior chamber of rabbit followed by Descemet’s membrane stripping, reported that it took four months to detect AQP1 immunoreactivity and see a significant reduction in corneal thickness and edema. These results suggest that the implantation of BM-MSCs on gelatin membranes [78] reduced the recovery time by two months compared to the injection of UC-MSCs alone [164]. This may be because Descemet’s membrane helps to keep the endothelial monolayer in place to maintain corneal clarity [164]. Although Descemet’s membrane was removed in the animal model in both studies, gelatin membranes may have acted as a substitute, thus reducing the recovery time. In line with this, Fei et al. [102] revealed that AM-MSCs injected into the corneal endothelium of cryoinjured mouse corneas stimulated the corneal microenvironment, led to corneal endothelial cell proliferation, promoting corneal damage repair, and increased mRNA expression levels of corneal endothelial markers ATP1A1 and ZO1, 10 days after transplantation. Considering the cryoinjury-induced desquamation of CEnCs while Descemet’s membrane remained intact, the reason for the reduced recovery time may be due to the membrane and not the cells. Therefore, in the study by Shao et al. [164], if the endothelium had been removed alone without Descemet’s membrane, the results might have been better.

On the clinical side, to our knowledge, no clinical trials using MSCs to regenerate corneal endothelium have been described, despite these outstanding results. This may be because, although MSCs can be differentiated into CEnC-like cells and have a similar shape and some similar characteristics to native CEnCs, differentiation may not be complete. This may present some drawbacks, such as very limited pump and barrier function of these cells, complications with respect to endothelial-mesenchymal transformation, or tumor formation. In addition, the few existing preclinical studies do not ensure the longevity of the graft [165].

### 4.2. Differentiated MSCs

Other authors revealed that endothelial recovery time was shorter if MSCs were differentiated into CEnC-like cells before transplantation. Shao et al. [166], who generated CEnC-like cells from BM-MSCs on a porcine corneal acellular matrix using hCEnCs coculture, improved the results obtained by Liu et al. [78], who implanted undifferentiated BM-MSCs on gelatin membranes. Shao et al. [166] reported a recovery of transparency and reduction of edema at 28 days after surgery instead of 2 months [78].

Hatou et al. [167], Inagaki et al. [64], and Yamashita et al. [168] induced corneal endothelial-like cell differentiation from human corneal stroma mesenchymal stem cells (CSSCs) [167], human skin-derived precursors (SKPs) [64], and UC-MSCs [168], respectively, with medium containing GSK-3β inhibitor and Rho-associated protein kinase inhibitor (Y-27632) on atelocollagen type I sheets for one week. Next, tissue-engineered corneal endothelium was transplanted into a rabbit model of corneal endothelial dysfunction. In the three studies, eight days post-operation, expression of the corneal endothelial pump marker Na^+^/K^+^ ATPase was upregulated, the corneas were less edematous, corneal thickness was significantly reduced, and corneal transparency was restored. These findings suggest that the recovery time using differentiated MSC-derived collagen tissue-engineered corneal endothelia depends on the type of stem cells used. CEnC-like cells from BM-MSCs showed worse results because they took longer (28 days) to show recovery [166] than CEnC-like cells from CSSCs, SKPs, or UC-MSCs, which took 8 days [64,167,168]. This suggests that the use of BM-MSCs differentiated into CEnCs on collagen sheets was not enough to shorten the recovery time. In addition, Shao et al. [166] used a human corneal endothelial cell coculture to induce CEnCs. Considering that the intention of using stem cell therapy is to find new functional CEnCs to replace hCEnCs, the use of hCEnC coculture does not solve the existing problem of corneal shortage.

Shen et al. [169] who injected CEnCs-like cells from SKPs co-cultured with hCEnCs observed, seven days post-surgery, that the corneas had become clearly transparent, corneal thickness had rapidly decreased and CEnC-like cells expressed Na^+^/K^+^ ATPase in a rabbit corneal endothelial dysfunction model.

They also injected the SKP-derived CEnCs in a monkey model of corneal dysfunction and reported that, during a two-year follow-up, the corneas remained transparent and normal in thickness. Cell density tended to be stable at above 2000 cells/mm^2^, in accordance with the normal physiological state, and CEnC-like cells continued to show Na^+^/K^+^ ATPase and collagen type VIII expression [155]. These results suggest that injecting SKPs-derived CEnCs by coculture with hCEnCs [169] produces the same results as using SKP-derived tissue-engineered corneal endothelia with GSK-3β inhibitor and Y-27632 [64]. Although Shen et al. [169] avoided the use of collagen sheets, they used hCEnC coculture to induce CEnC-like-cells, which is a disadvantage, as mentioned above.

Zhang et al. [170] and Ye et al. [171] injected UC-MSCs-derived CEnCs with Y-27632 in a rabbit model of corneal endothelial dysfunction and took three weeks [171] and six weeks [170] to see the recovery of corneal transparency. Considering that UC-MSCs-derived tissue-engineered corneal endothelia with GSK-3β and Y-27632 took eight days to show recovery [168], these findings suggest that the use of the GSK-3β inhibitor for differentiation and a collagen scaffold for transplantation is necessary to reduce recovery time with UC-MSC-derived CEnCs. This may be because collagen sheets not only help to keep the endothelial monolayer in place but also induce a better differentiation of the stem cells in CEnC-like cells, similar to the differentiation produced by hCEnC coculture. In this regard, our group has demonstrated the usefulness of human decellularized stroma as a carrier that can improve visual quality in a rabbit model of corneal endothelial disease in one month [68]. Moreover, the use of collagen imprints with Descemet-like topography (DLT) could further improve restoration time. It has been shown that differentiated Wharton jelly-derived MSCs on a collagen type I scaffold with DLT were able to maintain corneal transparency and increased expression of ZO1 and Na^+^/K^+^ ATPase after five days of cultivation in an ex vivo corneal endothelium-denuded rabbit cornea [172].

Based on these studies, the best strategy, as an alternative treatment for corneal endothelial deficiency, is the use of MSCs differentiated into CEnCs on collagen sheets with a medium containing GSK-3β inhibitor and Y-27632. However, no clinical applications using these protocols have been reported. This may be because the MSCs used in preclinical models have some drawbacks. The proliferative and differentiation potential of MSCs obtained from adult tissues, such as BM-MSCs and SKPs, depend on the donor’s age. Therefore, clinical application of autologous MSCs obtained from adult tissues may prove difficult when the source of cells is an elderly donor [168]. Furthermore, their harvesting requires an invasive procedure and does not yield a large number of stem cells. By contrast, UC-MSCs express pluripotency markers that are not present in adult stem cells, the stem cell isolation process is non-invasive, and the cell yield is not dependent on donor age [115]. Nevertheless, ADSCs, which can be easily obtained from minimally invasive liposuction aspirates, are superior in terms of the availability and number of multipotent stem cells obtained and have a higher proliferation capacity than UC-MSCs [173]. Moreover, ADSCs show more genetic and morphologic stability in long-term cultures [115].

**Figure 5:**
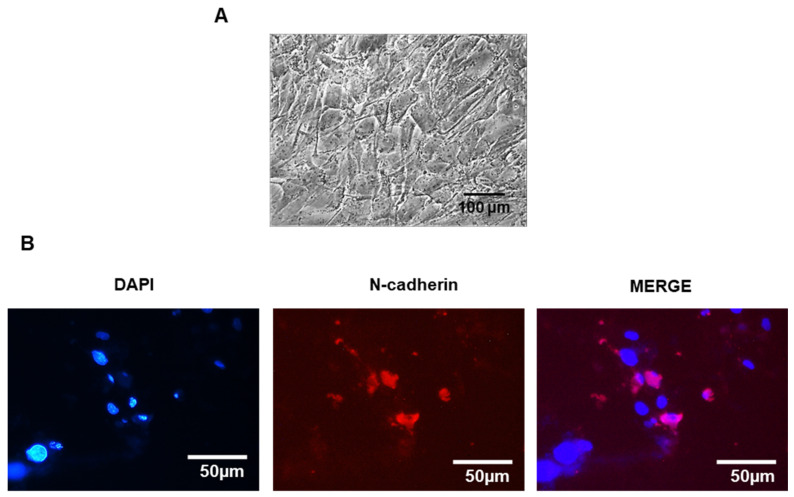
(**A**). Phase-contrast image of ADSC-derived CEnCs after 48 days. Note slight hexagonal morphology. (**B**). Confocal images of N-cadherin immunofluorescence (red) in cultivated human ADSC-derived CEnCs using Ali et al.’s differentiation media. DAPI nuclear staining in blue. Scale bars: 100 and 50 µm as indicated.

Taking into account the clear advantages of ADSCs and the fact that CEnCs are derived from the neural crest, our group investigated an in vitro method to obtain CEnCs using a two-step protocol to differentiate human ADSCs into CEnCs. First, human ADSCs were derived into neural crest stem-like cells with a neural induction medium, after which we adapted the protocols described by Ali et al. [174] and Wagoner et al. [175] for iPSC differentiation into CEnCs to induce CEnC differentiation. We demonstrated that within a month, ADSC-derived CEnCs increased Na^+^/K^+^ ATPase and cadherin expression (Figure 5) [176]. Bosch et al. [177] also used a different two-step differentiation protocol to induce DPSCs into CEnC-like cells. They showed that at day 12 of differentiation, such CEnCs differentiated into polygonal-like cells expressing upregulated Na^+^/K^+^ ATPase, ZO1, and collagen type VIII markers. These outcomes suggest that this differentiation protocol is faster with DPSCs than with ADSCs. However, unlike our research using defined media, Bosch et al. [177] used a primary human CEnC-conditioned medium. The use of CEnC-conditioned medium is likely to have contributed to the reduction in differentiation time, but its use, as with the coculture with CEnCs, is a disadvantage. Nevertheless, both methods provide a new pathway to explore the possibility of inducing CEnCs useful for future in vivo applications.

On the other hand, some researchers believe that research should focus on finding optimal protocols to expand hCEnCs while maintaining their function as an alternative treatment of corneal endothelial dysfunction. In this context, Peh et al. [65] described a successful approach for the propagation of primary hCEnCs using a dual media based on a proliferative medium followed by a maintenance medium. In addition, Peh et al. [178] and our group [68] demonstrated that hCEnCs cultured with the dual media on human decellularized corneal stroma sheets are able to reduce edema and increase corneal transparency within four weeks in a rabbit model of endothelial dysfunction.

Regarding the use of MSCs to propagate actual hCEnCs, Nakahara et al. [179] reported that after five days in the presence of conditioned medium obtained from human BM-MSCs (BM-MSCs-CM), hCEnCs not only stimulate proliferation but also maintain the characteristic differentiated phenotypes (ZO1 and Na^+^/K^+^-ATPase) required for the endothelial functions. With these findings, Sun et al. [165] injected expanded hCEnCs using human orbital ADSC-derived conditioned medium (OADSC-CM) into a rabbit model of corneal endothelial dysfunction and demonstrated that after seven days, transparency was restored, and corneal thickness decreased. Furthermore, they reported that after 10 months in the same study but using a longer-term monkey model, the corneas remained transparent, and the corneal thickness was normal as in healthy cornea, and Na^+^/K^+^ ATPase and ZO1 hCEnCs markers were still being expressed. Taking into account the good results obtained with MSC-conditioned medium as a tool to promote the proliferation of CEnCs, Buono et al. [180] and Ryu et al. [181] went a step further and evaluated the therapeutic effect of mesenchymal stem cell-derived extracellular vesicles (MSCs-EVs) in corneal endothelial dystrophy. Both demonstrated that human MSC-EVs were able to increase the viability of CEnCs by preventing cell apoptosis and restoring corneal endothelial damage in vitro [180] and in vivo in rats [181]. The study by Ryu et al. [181] has a number of advantages. They used MSCs-EVs instead of a conditioned medium, thus avoiding components of the medium that could pose a risk of immune rejection, such as the fetal bovine serum used by Sun et al. [165]. Moreover, as they did not transplant cells, the risk of cellular reactions was diminished, and therapeutic preparations were easier without having to consider cell survival issues. However, the corneal endothelial dysfunction model used in this research is a cryoinjured rat. This indicates that non-affected CEnCs may still have been present, which, stimulated by MSCs-EVs, proliferated and regenerated the corneal endothelium. Thus, it is not a good model of corneal endothelial dysfunction resembling actual patients, as both rat and rabbit endothelial cells are capable of proliferating in vivo in adults. in contrast to human CEnCs.

From a clinical point of view, based on the aforementioned studies and taking into account that, to our knowledge, no human studies have been published according to the official clinical trials website (Clinicaltrials.gov. Available online: https://clinicaltrials.gov/ (accessed on 24 October 2024)), MSCs differentiated into CEnCs could be used as an alternative treatment for corneal endothelial deficiency, as they have proven to be a good substitute for CEnCs. In addition, it may be possible to induce hCEnC proliferation in vivo with EVs carrying specific molecules, a pathway still to be investigated.

## 5. Conclusions and Future Perspectives

In the last decade, cell therapy with MSCs of different origins has been gaining momentum for corneal repair and regeneration. This is true for the three layers of the cornea, for which studies have moved from preclinical to clinical trials, and hopefully, pilot studies will be moving to phase II efficacy clinical trials. New advances in the understanding of corneal cell proliferation and function [182,183] and microenvironment [184] will definitely have an impact on future treatments in ophthalmology. In the near future, transplantation of MSCs together with hydrogels for the epithelium [185], stroma [10,186], and endothelium [187] will be the road to follow. In addition, iPSCs are also gaining clinical relevance [188] and, furthermore, the possibility of delivering drugs through nanoparticles [189] will surely impact many ophthalmological conditions and patients.

## Figures and Tables

**Table 1:** Examples of different modes of delivery of mesenchymal stem cells (MSCs) either as isolated cells or with carriers/scaffolds for corneal diseases and their advantages.

	Delivery Mode	Advantages	References
Isolated cells	Extracellular vesicles (EVs) or Exosomes	Replicates the regenerative effects of their parent cells Suppress the activation of immune cells Expression of proinflammatory factors	[30,32,33,34,35,36,37,38,39,40]
	Topical administration	Non-invasive, easy to apply Reduces the risk of surgical complications	[41,42,43,44,45]
	Intravenous injection	Enables systemic distribution	[46,47]
	Subconjunctival injection	Minimally invasive and easy to perform Allows the administration of high cell doses	[39,40,41,42,44,48,49,50]
	Intraperitoneal injection	Increased drug absorption Ensured the survival of MSCs for 3–4 weeks	[51,52]
	Intralesion injection: intralimbal, intrastromal	Decreased healing time and post-injury haze	[53,54,55,56]
Tissue engineering scaffolds/carriers	Amniotic membrane (AM)	Composition rich in collagen I and III mimics that of the extracellular matrix of the limbal niche Translucent Favorable mechanical properties such as elasticity and cell adhesion Anti-inflammatory, antimicrobial, and angiogenic properties	[26,27,50,57,58,59,60,61,62,63]
	Collagen	Provides a biosimilar substitute Avoids rejection and allows better cell survival	[64]
	Decellularized corneal stroma	Provides the components of the corneal extracellular matrix that mimics the mechanical properties of the cornea	[6,65,66,67,68]
	Silk membranes	Enhanced nutrient diffusion and cellular interaction Transparent and mechanically robust material Controllable biodegradation	[69,70]
	Fibrinogen/ Fibrin-agarose	Low cost Good tolerance and adhesion of cells	[28,29,71,72,73,74]
	Chitosan	Biodegradable, biocompatible, antibacterial, and antifungal Easy to manufacture Promotes wound healing	[30,75]
	Hyaluronic acid	Promotes the proliferation of MSCs inducing an anti-inflammatory profile	[39,76]
	Polysaccharide from Hardy Orchid	Low cost Promotes the recovery of burn injury	[77]
	Gelatin	High collagen content	[78]
	Poly (ethyl acrylate) (PEA)	Promotes fibronectin organization into nano-networks Enables MSCs to maintain self-renewal and functional multipotency Biointegration is improved when used with natural coatings	[79,80]
	Polylactic acid (PLA) nanofibers	Good mechanical strength, hydrophilicity, thermal plasticity, and porosity Promotes cell adhesion and growth Degraded into nontoxic by-products that are eliminated through the urine	[42,75,81]

**Table 2:** Clinical trials using mesenchymal stem cells (MSCs) or MSCs-derived exosomes (exo-MSCs) to regenerate corneal epithelium with published results, most of them registered in ClinicalTrials.gov. Available online: https://clinicaltrials.gov/ (accessed on 24 October 2024). Abbreviations: ADDE: aqueous-deficient dry eye disease; DED: dry eye disease; GVHD: graft-versus-host disease; LSCD: limbal stem cell deficiency; SS: Sjögren’s syndrome.

ClinicalTrials.gov Number/Reference	Target Disease	MSCs Source	Mode of Administration	Number of Patients	Follow-Up
Weng et al. [46]	GVHD-DED	Allogeneic bone marrow	Intravenous injection	22	3 months
NCT01562002 Calonge et al. [61]	LSCD	Allogeneic bone marrow	On amniotic membrane transplant	27	12 months
NCT03878628 Møller-Hansen et al. [119]	SS-ADDE	Allogeneic adipose tissue	Injected into the lacrimal gland	7	4 months
NCT04213248 Zhou et al. [38]	GVHD-DED	Umbilical mesenchymal stem cells-derived exosomes	Eye drops	27	3 months
NCT04615455 Møller-Hansen et al. [120]	SS-ADDE	Allogeneic adipose tissue	Injection into the lacrimal gland	54	12 months
NCT01808378 Boto de Los Bueis et al. [50]	LSCD	Autologous adipose tissue	Injection into the limbus conjunctiva + Eye drops	8	86.5 months

**Table 3:** Clinical trials using mesenchymal stem cells (MSCs) to regenerate corneal stroma registered in ClinicalTrials.gov. Available online: https://clinicaltrials.gov/ (accessed on 24 October 2024).

ClinicalTrials.gov Number/Reference	Disease	MSCs Source	Mode of Administration	Number of Patients	Follow-Up
NCT03237442	Corneal burn	Allogeneic umbilical cord	Subconjunctival injections	100	3 months
NCT02932852 El Zarif et al. [6]	Advanced keratoconus	Autologous adipose tissue	Intrastromal implantation	14	3 years
NCT02948023	Corneal wound, corneal burn	Allogeneic limbal stromal stem cells	Topically onto the ocular surface embedded into a fibrin gel	100	3 months
NCT03295292 Basu et al. [145]	Corneal scarring	Allogeneic limbal stromal stem cells	Topically onto the ocular surface embedded into a fibrin gel	15	2 years
NCT05279157	Stromal corneal dystrophies	Autologous adipose tissue	Implantation with or without scaffold in the intrastromal cornea	15	12 months

**Table 4:** Published results of the clinical trial NCT02932852.

Study Reference	Disease	MSCs Source	Method	Results	Follow-Up
Alió Del Barrio et al. [146]	Advanced keratoconus	Autologous adipose tissue	Intrastromal injection of autologous ADSCs (3 × 10^6^ cells) in 14 patients using a femtosecond-assisted lamellar pocket	Improvements in corneal transparency and visual acuity, without complications. Formation of new collagen confirmed by confocal microscopy.	6 months
Alió et al. [67]	Advanced keratoconus	Autologous adipose tissue	Comparative implantation of ADSCs alone (*n* = 5), decellularized corneal stroma (*n* = 5), or ADSCs with decellularized laminae for structural support in the cornea (*n* = 4)	Significant improvements in corneal thickness and transparency and visual acuity across the three experimental groups. All groups demonstrated improved structural integrity.	1 year
El Zarif et al. [144]	Advanced keratoconus	Autologous adipose tissue	Report on 3-year outcomes of ADSCs and decellularized or ADSCs-recellularized corneal laminae	Significant increases in corneal thickness and topographic stability. No complications, and stable increase in corneal thickness, visual acuity improvement	3 years

## Data Availability

No new data were created.
